# The Role of Saccharides in the Mechanisms of Pathogenicity of *Fusarium oxysporum* f. sp. *lupini* in Yellow Lupine (*Lupinus luteus* L.)

**DOI:** 10.3390/ijms21197258

**Published:** 2020-10-01

**Authors:** Magda Formela-Luboińska, Dorota Remlein-Starosta, Agnieszka Waśkiewicz, Zbigniew Karolewski, Jan Bocianowski, Łukasz Stępień, Mateusz Labudda, Philippe Jeandet, Iwona Morkunas

**Affiliations:** 1 Department of Plant Physiology, Poznań University of Life Sciences, Wołyńska 35, 60-637 Poznań, Poland; magda.formela-luboinska@up.poznan.pl; 2 Department of Ecology and Environmental Protection, Institute of Plant Protection—National Research Institute, Władysława Węgorka 20, 60-318 Poznań, Poland; 3 Department of Chemistry, Poznań University of Life Sciences, Wojska Polskiego 75, 60-625 Poznań, Poland; agnieszka.waskiewicz@up.poznan.pl; 4 Department of Phytopathology, Seed Science and Technology, Poznań University of Life Sciences, Dąbrowskiego 159, 60-594 Poznań, Poland; zbigniew.karolewski@up.poznan.pl; 5 Department of Mathematical and Statistical Methods, Poznań University of Life Sciences, Wojska Polskiego 28, 60-637 Poznań, Poland; jan.bocianowski@up.poznan.pl; 6 Department of Pathogen Genetics and Plant Resistance, Institute of Plant Genetics, Polish Academy of Sciences, Strzeszyńska 34, 60-479 Poznań, Poland; lste@igr.poznan.pl; 7 Department of Biochemistry and Microbiology, Institute of Biology, Warsaw University of Life Sciences-SGGW, Nowoursynowska 159, 02-776 Warsaw, Poland; mateusz_labudda@sggw.edu.pl; 8 Research Unit “Induced Resistance and Plant Bioprotection”, UPRES EA 4707, Department of Biology and Biochemistry, Faculty of Sciences, University of Reims, P.O. Box 1039, CEDEX 02, 51687 Reims, France; philippe.jeandet@univ-reims.fr

**Keywords:** ergosterol, fungal sporulation, *Fusarium oxysporum*, *Lupinus luteus*, moniliformin, mycelium growth, sugars

## Abstract

The primary aim of this study was to determine the relationship between soluble sugar levels (sucrose, glucose, or fructose) in yellow lupine embryo axes and the pathogenicity of the hemibiotrophic fungus *Fusarium oxysporum* f. sp. Schlecht *lupini*. The first step of this study was to determine the effect of exogenous saccharides on the growth and sporulation of *F*. *oxysporum*. The second one focused on estimating the levels of ergosterol as a fungal growth indicator in infected embryo axes cultured in vitro on sugar containing-medium or without it. The third aim of this study was to record the levels of the mycotoxin moniliformin as the most characteristic secondary metabolite of *F*. *oxysporum* in the infected embryo axes with the high sugar medium and without it. Additionally, morphometric measurements, i.e., the length and fresh weight of embryo axes, were done. The levels of ergosterol were the highest in infected embryo axes with a sugar deficit. At the same time, significant accumulation of the mycotoxin moniliformin was recorded in those tissues. Furthermore, it was found that the presence of sugars in water agar medium inhibited the sporulation of the pathogenic fungus *F*. *oxysporum* in relation to the control (sporulation of the pathogen on medium without sugar), the strongest inhibiting effect was observed in the case of glucose. Infection caused by *F*. *oxysporum* significantly limited the growth of embryo axes, but this effect was more visible on infected axes cultured under sugar deficiency than on the ones cultured with soluble sugars. The obtained results thus showed that high sugar levels may lead to reduced production of mycotoxins by *F*. *oxysporum,* limiting infection development and fusariosis.

## 1. Introduction

Sugars are essential nutrients for the development of fungal pathogens because these metabolites are used as carbon skeletons for the biosynthesis of other compounds as well as serving of substrates for energy production and being involved in cellular signaling pathways in fungi [1]. As reported by Jones and Dangl [2], access to nutrients of host plant cells, which allows fungal pathogens to colonize, develop, and reproduce, is the primary goal during their life cycle. Sucrose and monosaccharide transporters in host cells constitute the key components for carbon partitioning at the whole plant level as well as during the initiation of sugar flux towards the attacked tissues and upon interactions with fungi [3]. Moreover, cell-wall invertases cleaving sucrose into glucose and fructose in the apoplast of plant cells are key enzymatic regulators of carbon partitioning under fungal infection [4,5,6,7,8,9]. 

The research nowadays focuses on the mechanisms underlying exchange of sugars between fungi and their host plants. A significant role of fungal monosaccharide transporters has been suggested during these interactions [10]. The roles of hexose and sucrose transporters (SWEET), as well as sugar transport protein (STP) transporters mediating transport of sugars across the plasma membrane, have been widely described [11,12]. Fungal pathogens that damage cell walls and inject effectors during the biotrophic stage have the ability to modify the host plant cell metabolism causing redirection of sugars, increase sink strengths, and express invertases as well as sugar transporters to acquire carbon compounds [1]. Moreover, it has been shown that high levels of sugars may influence cell wall-degrading extracellular enzymes secreted by fungal pathogens. These enzymes may contribute to pathogenesis by degrading wax, cuticle, and cell walls, thus aiding in tissue invasion and pathogen dissemination [13,14]. Patil and Dimond [15] were the first to show that repression of polygalacturonase synthesis by sugars in *Fusarium oxysporum* affects symptom reduction in infected plants. 

As Bani et al. [16] reported, successful host invasion by *F. oxysporum* depends on a multitude of factors, which vary according to the pathosystem considered. During seed germination, particularly in the heterotrophic phase, both the adverse effects of environmental factors and deep sowing may disturb the distribution of metabolites reaching from source tissues, i.e., cotyledons, to sink tissues—the embryo axis of the germinating seed, which as a consequence may lead to reduced soluble sugar levels and increased susceptibility of germinating seeds to infections by *F. oxysporum* [17,18]. This pathogen is representative of soilborne pathogens inhabiting the soil for a long time in the form of chlamydospores, whose hyphae penetrate the roots, spreading in the tissues, colonizes and metastasizes in xylem vessels, and causes systemic wilting, as well as pre-emergent sprout root and post-emergent seedling rot and death in plants. 

It is well known that during plant and pathogen coevolution, the plant limits pathogen access to nutrients and initiates defense responses, whereas the pathogen evolves adaptive strategies to gain access to nutrients and suppress host immunity [11]. Thus, during the pathogen and plant coevolution, in each of these organisms, both gradual and mutual adjustment occurs because of a kind of feedback coupling. The phenomenon of fungal virulence, i.e., pathogenicity, is expressed by pathogen invasiveness (the ability of a pathogen to penetrate plant tissues, proliferate and spread) as well as toxicity (fungal ability to produce toxins) [19]. In view of the above, we first intended to answer the question, whether different levels of soluble sugars in *F. oxysporum*-infected yellow lupine embryo axes may have an impact on the ergosterol (ERG) level—an important sterol found in cell membranes of fungi and whose content in plant tissues is an indicator of the development of the fungal pathogen. Secondly, whether and which soluble sugars may regulate the production of moniliformin (MON)—a highly toxic secondary metabolite synthesized by *F. oxysporum*. Parallelly, we examined the effect of exogenous saccharides on the growth and sporulation (formation of both macroconidia and microconidia) of *F. oxysporum* f. sp. *lupini* in *in vitro* conditions. As a typical vascular wilt fungus, *F. oxysporum* produces a characteristic xylem vessel clogging and blocks the transport of water in the xylem while causing wilting of the infected plants. Therefore, it was important in this study to compare the plant’s biomass, especially the fresh weight of the embryo axes cultured under different trophic conditions, with high sugar levels and with sugar deficiency. Colonization and clogging of vessels, in addition to the secretion of several toxins by the fungus (fusaric acid, lycomarasmin, and dehydrofusaric acid), play a major role in wilt symptom development and progression [20]. As reported by Perincherry et al. [21], among the *Fusarium* mycotoxins, the primarily concerned ones are the trichothecenes, fumonisins, and zearalenones. *F*. *oxysporum* is mainly recognized as a moniliformin-producing fungus [22,23], and its mycotoxin has the potential to inhibit plant growth [21]. It has been proved that some *F*. *oxysporum* strains can also biosynthesize fumonisins B_1_–FB_1_ [24,25,26].

Our hypothesis assumes that high levels of sucrose and monosaccharides may lead to the reduction of *F. oxysporum* virulence and limitation of the infection development, as well as fusariosis. The phytopathogenic fungus *F. oxysporum* f. sp. *lupini* is a hemibiotrophic fungus that combines two attack strategies. In the initial biotrophic phase, during which the host’s immune system and cell death are actively suppressed, invasive hyphae spread throughout the infected plant tissues. In the next phase, the fungus secretes toxins and enzymes that kill host cells, and then the fungus takes up the nutrients released from the dead tissues. Therefore, a detailed understanding of the role of sugars and their effects on the pathogenicity of this fungus will contribute to a better understanding of the mode of action of a fungal pathogen under different trophic conditions.

## 2. Results

### 2.1. Effects of Saccharides on the Growth and Sporulation of F. oxysporum f. sp. lupini 

**Figure 1:**
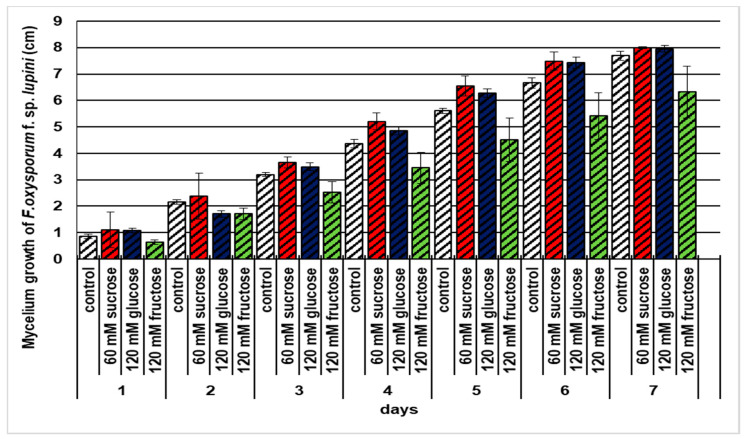
Effects of sucrose, glucose, and fructose on the mycelium growth of *Fusarium oxysporum* f. sp. *lupini*. A summary of the statistical significance of differences between the average values of each pair at *p* < 0.05 can be found in the Supplementary Files (Table S1).

Analysis of the linear growth of the mycelium of *F*. *oxysporum* f. sp. *lupini* on water agar showed that exogenous administration of soluble carbohydrates, i.e., 60 mM sucrose and 120 mM glucose, slightly stimulated the growth of this fungus. However, 120 mM fructose clearly inhibited pathogen growth relative to control (water agar without sugar) (Figure 1). 

**Figure 2:**
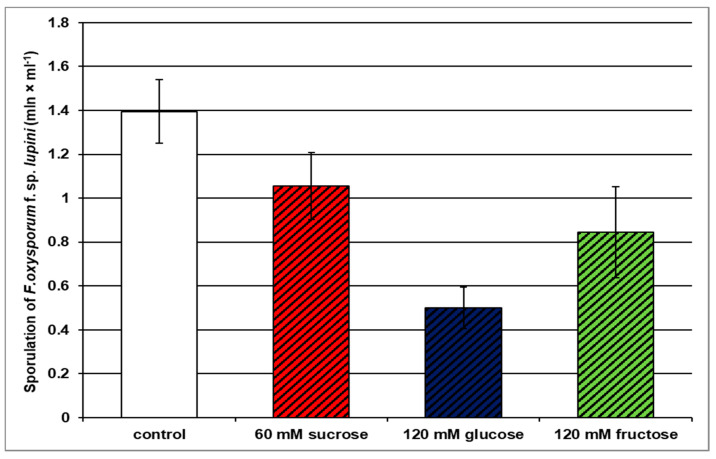
Effects of sucrose, glucose, and fructose on the sporulation (formation of both macroconidia and microconidia) of *Fusarium oxysporum* f. sp. *lupini*. A summary of the statistical significance of differences between the average values of each pair at *p* < 0.05 can be found in the Supplementary Files (Table S2).

In addition, both 60 mM sucrose, 120 mM glucose, and 120 mM fructose were found to inhibit *F*. *oxysporum* sporulation (formation of both macroconidia and microconidia) (Figure 2). It should be emphasized that among tested soluble carbohydrates, glucose inhibited the fungus sporulation most strongly. The sporulation of *F*. *oxysporum* on the glucose medium was about 3% less effective than the sporulation of the pathogen on the medium without sugar (control). In turn, sucrose inhibited sporulation by 27% relative to control, while fructose by 42% (Figure 2).

### 2.2. Effects of Saccharides on the Accumulation of Moniliformin in Embryo Axes of L. luteus Infected with F. oxysporum f. sp. lupini

**Figure 3:**
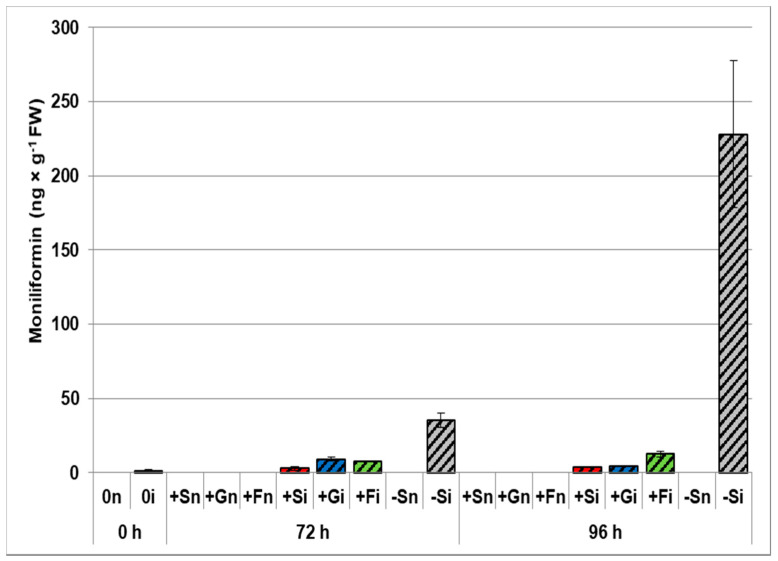
Effects of sucrose, glucose, and fructose on the accumulation of moniliformin in *in vitro* cultured embryo axes of *Lupinus luteus* infected with *Fusarium oxysporum* f. sp. *lupini*. (+Sn, non-inoculated embryo axes and cultured in vitro on Heller’s medium with 60 mM sucrose; +Gn, non-inoculated embryo axes and cultured in vitro on Heller’s medium with 120 mM glucose; +Fn, non-inoculated embryo axes and cultured in vitro on Heller’s medium with 120 mM fructose; −Sn, non-inoculated cultured in vitro on medium without sucrose; +Si, inoculated and cultured with 60 mM sucrose; +Gi, inoculated and cultured with 120 mM glucose; +Fi, and with 120 mM fructose; −Si, inoculated and cultured without sucrose). A summary of the statistical significance of differences between the average values of each pair at *p* < 0.05 can be found in the Supplementary Files (Table S3).

Experiments showed a significant accumulation of moniliformin in infected embryo axes of yellow lupine grown on sugar-free medium (Figure 3). Very high production of this mycotoxin was recorded at 72 and 96 h after infection in the embryo axes of lupine grown without sugars, the level of this mycotoxin being 35 ng × g^−1^ fresh weight (FW) and 228 ng × g^−1^ FW, respectively. Additionally, at all time points after infection, a significantly lower moniliformin production was found in embryo axes cultured on sucrose, glucose, or fructose medium than on embryo axes cultured without sugars. For 72 h embryo axes cultured on a medium with 60 mM sucrose (+Si), 120 mM glucose (+Gi), and 120 mM fructose (+Fi), the levels of this mycotoxin were 3.12, 9.24, and 7.75 ng × g^−1^ FW, respectively. In turn, in 96 h axes grown on a medium with sucrose (+Si), glucose (+Gi), or fructose (+Fi), the moniliformin contents were 3.72, 4.29, and 12.57 ng × g^−1^ FW, respectively, while they were many times higher in −Si axes compared to all other experimental variants.

### 2.3. Effects of Saccharides on the Accumulation of Ergosterol in Embryo Axes of L. luteus Infected with F. oxysporum f. sp. lupini

**Figure 4:**
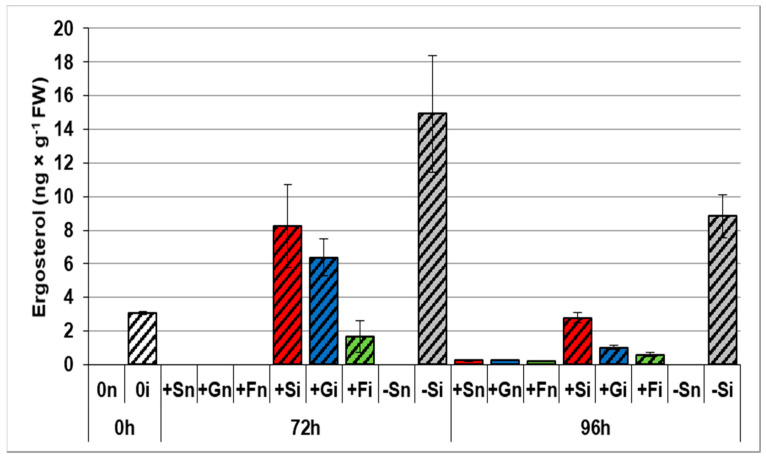
Effects of sucrose, glucose, and fructose on the accumulation of ergosterol in *in vitro* cultured embryo axes of *Lupinus luteus* infected with *Fusarium oxysporum* f. sp. *lupini*. (+Sn, non-inoculated embryo axes and cultured in vitro on Heller’s medium with 60 mM sucrose; +Gn, non-inoculated embryo axes and cultured in vitro on Heller’s medium with 120 mM glucose; +Fn, non-inoculated embryo axes and cultured in vitro on Heller’s medium with 120 mM fructose; −Sn, non-inoculated cultured in vitro on medium without sucrose; +Si, inoculated and cultured with 60 mM sucrose; +Gi, inoculated and cultured with 120 mM glucose; +Fi, and with 120 mM fructose; −Si, inoculated and cultured without sucrose). A summary of the statistical significance of differences between the average values of each pair at *p* < 0.05 can be found in the Supplementary Files (Table S3).

The ergosterol level, as a fungal growth indicator, was found to be significantly higher in embryo axes infected with *F*. *oxysporum* and cultured without sugars than in infected embryo axes cultured with sugars (Figure 4). The highest content of ergosterol in inoculated embryo axes cultured under a sugar deficit (−Si) was recorded at 72 h: 14.92 ng × g^−1^ FW. On the other hand, embryo axes cultured on the medium with sugars contained the lowest levels of ergosterol, especially those grown on the 120 mM fructose medium.

### 2.4. Effects of Saccharides and F. oxysporum f. sp. lupini Infection on the Growth of Yellow Lupine Embryo Axes

**Figure 5:**
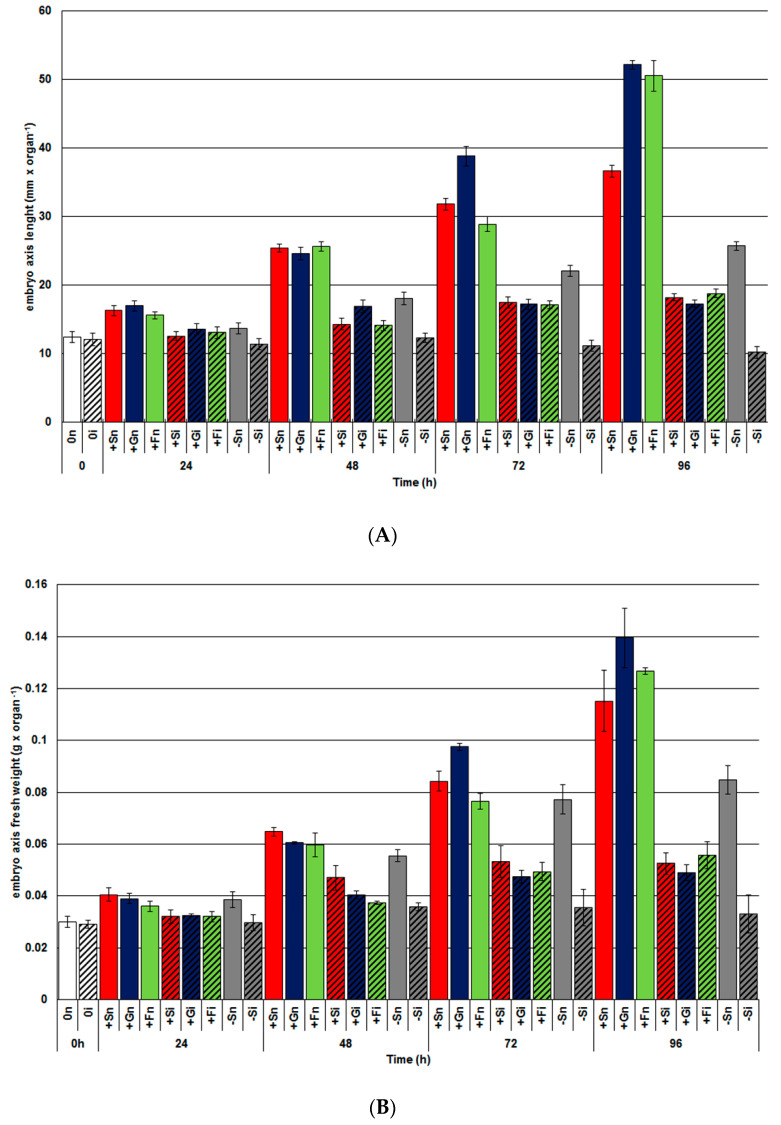
Effects of sucrose, glucose, and fructose on the growth ((**A**) length and (**B**) fresh weight) of cultured in vitro embryo axes of *Lupinus luteus* infected with *Fusarium oxysporum* f. sp. *lupini*. (+Sn, non-inoculated embryo axes and cultured in vitro on Heller’s medium with 60 mM sucrose; +Gn, non-inoculated embryo axes and cultured in vitro on Heller’s medium with 120 mM glucose; +Fn, non-inoculated embryo axes and cultured in vitro on Heller’s medium with 120 mM fructose; −Sn, non-inoculated cultured in vitro on medium without sucrose; +Si, inoculated and cultured with 60 mM sucrose; +Gi, inoculated and cultured with 120 mM glucose; +Fi, and with 120 mM fructose; −Si, inoculated and cultured without sucrose). A summary of the statistical significance of differences between the average values of each pair at *p* < 0.05 can be found in the Supplementary Files (Table S4).

Morphometric measurements of yellow lupine embryo axes cultured in vitro on Heller medium supplemented with 60 mM sucrose, 120 mM glucose, and 120 mM fructose showed that saccharides alone strongly stimulated the growth of axes, i.e., an increase in both the length and the biomass versus cultivation time (from 0 to 96 h) (Figure 5A,B). Besides, among soluble sugars, glucose most strongly stimulated the length and fresh weight of embryo axes (Figure 5A,B). Moreover, under sugar starvation conditions, non-inoculated embryo axes (−Sn) underwent increased growth versus time though this was significantly smaller than that observed with embryo axes cultured with saccharides (+Sn, +Gn and +Fn). Infection caused by *F*. *oxysporum* very strongly limited the length and the fresh weight of embryo axes, but more definitely for those cultured under sugar deficiency (−Si) compared to axes with high sugar levels (+Si, +Gi, and +Fi). At 72 and 96 h, the length and fresh weight of embryo axes cultured on the medium with sucrose, glucose, and fructose (+Si, +Gi, +Fi) were higher than in inoculated axes with sugar starvation (−Si).

### 2.5. Correlation Analysis

**Figure 6:**
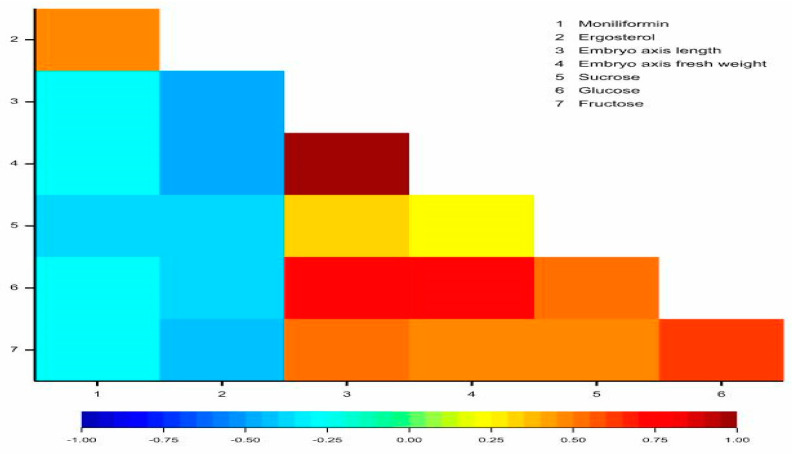
Heatmap matrix for linear Pearson’s correlation coefficients between moniliformin, ergosterol, embryo axis length, embryo axis fresh weight, sucrose, glucose, and fructose (r_cr_ = 0.2686).

A positive correlation was observed between the following pairs of studied traits: moniliformin and ergosterol (r = 0.4714), embryo axis length and embryo axis fresh weight (r = 0.9604), embryo axis length and sucrose (r = 0.3098), embryo axis length and glucose (r = 0.7707), embryo axis length and fructose (r = 0.5358), embryo axis fresh weight and glucose (r = 0.7164), embryo axis fresh weight and fructose (r = 0.4868), sucrose and glucose (r = 0.5214), sucrose and fructose (r = 0.4547), as well as glucose and fructose (r = 0.6206) (Figure 6). Negative significant relationships were observed between: moniliformin and embryo axis length (r = −0.2931), moniliformin and embryo axis fresh weight (r = −0.2783), moniliformin and sucrose (r = −0.3552), ergosterol and embryo axis length (r = −0.4596), ergosterol and embryo axis fresh weight (r = −0.4539), ergosterol and sucrose (r = −0.3867), ergosterol and glucose (r = −0.3638), and ergosterol and fructose (r = −0.4089) (Figure 6).

**Figure 7:**
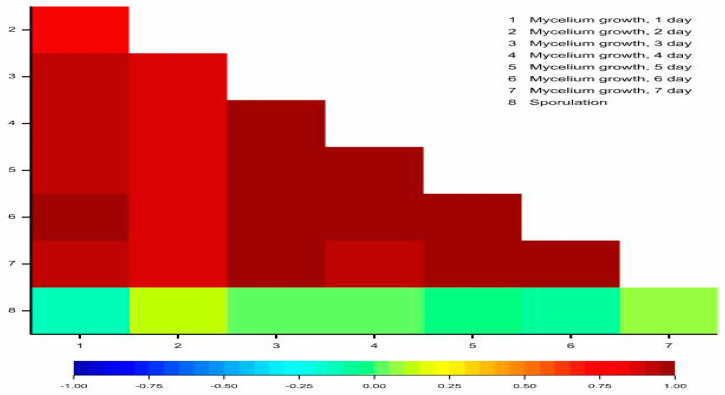
Heatmap matrix for linear Pearson’s correlation coefficients between mycelium growth (observed in 1–7 days) and sporulation (r_cr_ = 0.3125).

We observed statistically significant correlation coefficients for mycelium growth in all days of the study (Figure 7). The sporulation values were not correlated with mycelium growth (Figure 7).

## 3. Discussion

The results of this study show, for the first time, a reduction of the pathogenicity of *F. oxysporum* f. sp. *lupini* in the pathosystem constituted by this hemibiotrophic fungus and yellow lupine embryo axes. Experiments reported that ergosterol (ERG) accumulation, the major product of sterol biosynthesis in fungi and an indicator compound for estimating the content of fresh fungal biomass in plant tissues, was limited within the first 96 h after *F. oxysporum* infection by exogenous addition of sucrose, glucose or fructose to the medium (Figure 4). At the same time, our results clearly showed that accumulation of moniliformin (MON), a characteristic mycotoxin produced by *F. oxysporum*, was significantly lower in embryo axes cultured on a saccharide-containing medium (e.g., in +Si axes) than in those cultured under sugar starvation (–Si) (Figure 3). For example, the levels of MON were respectively 11 and 61 times higher in infected -Si embryo axes than in +Si embryo axes at 72 and 96 h, respectively. It has become clear that sugar starvation causes a horrendous increase in the sensitivity of embryo axes to pathogenic fungus *F. oxysporum*, which leads to tissue death, as it was demonstrated by our group in a previous study conducted with the same model system [27]. We have previously shown that *F. oxysporum* caused dieback of infected *L. luteus* L.cv. Juno embryo axes cultured on the medium without sugar (–Si), while disease symptom intensity occurring in infected embryo axes cultured on a medium with sugar (+Si, +Gi or +Fi) was limited. Also, our earlier research on a different variety of lupine, i.e., *L. luteus* L. cv. Polo (a variety sensitive to fusariosis) infected with *F. oxysporum* f. sp. *lupini* revealed that both the infection and the development of fusariosis in +Si embryo axes was inhibited in comparison to –Si embryo axes [28], demonstrated as shown by through ultrastructure examinations, reporting that fungal hyphae occurred in intracellular spaces of –Si embryo axes, but did not in the case of intracellular spaces of +Si embryo axes. 

It should be emphasized that hitherto research with *F. oxysporum* focused on embryo axes of yellow lupine, however, analyses were also done in *in vitro* conditions in order to obtain comprehensive information regarding the effect of exogenous soluble sugars on the growth and sporulation (formation of macroconidia and microconidia) of pathogenic fungus *F. oxysporum* f. sp. *lupini*. Our results clearly show an inhibition of the sporulation of this pathogen by soluble sugars (the culture of the pathogen on water agar medium with sugar), especially glucose, thereby suggesting that soluble sugars can play an important role in limiting the development and spread of systemic pathogens such as *F. oxysporum*. Over the past 20 years, research conducted by Morkunas and co-workers, has demonstrated the protective role of sucrose in plant tissues towards fungal pathogens. It has been postulated from those studies that high levels of sucrose enable plants to stimulate efficient defense mechanisms against *F. oxysporum* [28,29,30,31,32,33,34]. For example, sugars play a key role in plant defense responses to infection caused by fungal pathogens [35,36,37]. First, they are the main substrates utilized in respiration processes supplying energy for cellular defense responses against pathogens [28,32,38,39]. Moreover, it has been documented that sugars provide the carbon skeleton for the synthesis of defense compounds, including secondary metabolites such as flavonoids [29,31], lignins [30], and stilbenes [40,41]. Finally, carbohydrates, such as sucrose, glucose, and fructose, represent also metabolic signaling molecules in host plant cells that induce the expression of many genes, including defense genes [33,42,43,44,45].

It should be mentioned that Fusaria are fungal pathogens which produce a high level of mycotoxins and their ability for the simultaneous biosynthesis of multiple metabolites from different metabolic pathways is well-known [46]. Moreover, it has been reported that mycotoxin biosynthesis in the *Fusarium* genus is dependent on many environmental factors such as pH, temperature, moisture content, and nitrogen source [21]. The pathogenic fungus *F. oxysporum* is recognized mainly as a moniliformin producer. MON, naturally occurring as the sodium or potassium salt of 3-hydroxycyclobut-3-ene-1,2-dione, is a highly toxic secondary metabolite to plants though, fumonisin B_1_ has also been detected in some *F. oxysporum* strains [22,23]. These mycotoxins cause different toxicological effects in both humans and animals [47]. The feeds contaminated with mycotoxins have been shown to cause a number of mycotoxicoses, including leukoencephalomalacia in horses, pulmonary edema in swine, altered hepatic, and immune function in cattle as well as liver cancer and neural tube defects in experimental rodents [22,48]. 

Another type of direct limiting influence of sugar on the virulence of fungal pathogens is the effect of host plant carbohydrates on fungal metabolism. The high level of sugar may lead to disturbances in metabolic mechanisms of the pathogen, which limits its invasion of plant tissues. This is exemplified by the results of studies of the influence of sugar on cell wall-degrading extracellular enzymes secreted by fungal pathogens. Patil and Dimond [15] were the first to demonstrate that inhibition of polygalacturonase synthesis by carbohydrates in *F. oxysporum* reduces symptoms in infected tomato plants. In further studies, a special construct was used in which the green fluorescent protein (GFP) was fused as a reporter gene with the polygalacturonase promoter gene of the fungal pathogen. This approach made it possible to follow the expression of the polygalacturonase gene during infection as well as to determine the effect of various factors administered during incubation on its expression [49,50,51]. Studies using GFPs to detect the expression of the citrus plant pathogen *Alternaria citri* polygalacturonase gene, revealed a green fluorescence when pectins were present in the pathogen growing medium. However, when 1% glucose was added to the pectin medium, no fluorescence was observed, indicating that polygalacturonase gene expression was regulated by catabolic repression as it is well-known that a number of genes can be repressed in the presence of carbon sources such as glucose [52]. Otherwise, the pathogenic fungus *Botrytis cinerea* produces endopolygalacturonases which are involved in the degradation of pectins from the plant cell wall. Endopolygalacturonase isoenzymes in *B. cinerea* are encoded by as many as six genes (*Bcpg1*-*Bcpg6*), the expression of which can be controlled by various external factors [53]. For example, a low pH of the culture medium induces expression of the *Bcpg3* gene. The expression of the *Bcpg4* gene was shown to be repressed in the presence of carbon sources such as glucose. Wubben et al. [53] postulated that the presence of large gene families encoding cell wall-degrading enzymes is related to the host range of the fungus. *B. cinerea* is the only species of the genus Botrytis that has a broad host range. The other species are all confined to one host species or to a small plant genus. Later research showed that host pH and sugars are involved in the regulation of the genes encoding cell wall-degrading enzymes in many plant pathosystems [54]. Carbon catabolite repression of fungal polygalacturonase (PG) has been reported for *F. oxysporum* f. sp. *lycopersici* [15], *Aspergillus niger* [55], *Aspergillus nidulans* [56], *Cochliobolus carbonum* [57], and *Penicillium expansum* [58].

Moreover, another effect of the presence of carbohydrates in the host plant cell is the lowering of the host cell water potential, inhibiting growth and development of fungi [59]. Smith et al. [60] have shown that germination of spores and subsequent spore development, as well as growth of the hyphae of *F. oxysporum* were often delayed up to 72 h upon spore transfer from water agar to agar medium containing D-glucose or sucrose. At a concentration greater than 0.1 M, glucose levels in the medium usually inhibit the growth of the fungus. However, mono- and disaccharides are probably not the primary causes of the growth inhibition of *F. oxysporum* or other *Fusarium* species. Instead, catabolite repression involving the effect of high glucose concentrations on respiratory enzymes [61] or a deficiency or lack of an essential metabolite in spores or hyphal tips conditioned by high concentrations of glucose, seem to be some of the possible causes of the temporary growth inhibition [60].

It should be mentioned, however, that in the published literature, there are also studies reporting that sugars at a certain concentration can stimulate spore germination of some fungi, as demonstrated in *in vitro* tests. For example, it was proven that the addition of 0.5% sucrose to the medium stimulated spore germination in some species, such as *Aspergillus niger* and *Colletotrichum gloeosporioides* [62]. Moreover, the growth rate of *Pestalotia psyddii* was found to be greater in the presence of sucrose and fructose than in the presence of maltose, but lower than in controls [63]. In turn, Sangeetha and Rawal [64] proved that fructose is the best carbon source for the growth and sporulation of some *C. gloeosporioides* isolates that cause anthracnosis in papaya. In addition, mannitol and lactose caused poor sporulation of the isolates. No sporulation was also observed in the case of isolates growing on a medium without a carbon source. Xerophilic fungi such as *Eurotium* spp. are known to be resistant to external factors, including high sugar and salt concentrations [65]. Antony-Babu and Singleton [66] suggested a protective role of sucrose by showing a positive relationship between sucrose concentration (10–20%) and the ability to form conidia under the influence of ozone.

Summing up, the recorded results indicate that high sugar levels may lead to a reduced virulence of *F. oxysporum* f. sp. *lupini* through the limitation of mycotoxin production, infection and fusariosis development, which was manifested in the low level of the fungal indicator ergosterol. Additionally, the results of *in vitro* studies confirm the effect of sucrose and its related monosaccharides in inhibiting *F. oxysporum* sporulation (production of both macroconidia and microconidia).

## 4. Materials and Methods

### 4.1. Plant Material and Growth Conditions 

Seeds of yellow lupine (*Lupinus luteus* L. cv. Juno) were obtained from the Plant Breeding Company at Tulce near Poznań (Poland). The used cultivar is resistant to fusariosis according to breeding tests performed by the Plant Breeding Company in Poznań, Poland. Firstly, seeds were surface-decontaminated, and next immersed in sterile water and incubated at 25 °C. After six hours of imbibition, the seeds were put onto filter paper (in Petri dishes) and immersed in a small amount of sterile water. After the next eighteen hours, the seed coats were stripped out, and the cotyledons were dissected to isolate the embryo axes. At the beginning of the study (0 h), the embryo axes were inoculated with a *F. oxysporum* f. sp. *lupini* spore suspension, or they were not inoculated. The embryo axes were put, within the next twenty minutes after cotyledon dissection, in groups of four onto Whatman filter papers, which were afterward transferred to sterile glass tubes (diameter 3 cm, height 13.5 cm) containing fourteen milliliters of Heller’s mineral medium [29]. Eight culture conditions were used: +S, embryo axes cultured in vitro on Heller’s medium supplemented with 60 mM sucrose [17,27,28,29,30,32,33,34,38], +G, 120 mM glucose, and +F, 120 mM fructose [34], and −S, embryo axes cultured in vitro on Heller’s medium without sucrose (−Sn, non-inoculated cultured without sucrose and −Si, inoculated cultured without sucrose). Furthermore, axes before being transferred to glass tubes with Heller’s medium were not inoculated (the control, i.e. +Sn, +Gn and +Fn) or inoculated with *F. oxysporum* f. sp. *lupini* (+Si, +Gi and +Fi).

The used sucrose, glucose, and fructose concentrations were optimal to guarantee proper growth of the embryo axes, fresh and dry weight, as well as the uptake of nutrients from the medium (this was established on the basis of earlier tests) [34]. This model system is an equivalent of the stage in the development of germinating lupine seeds before the developing seedling emerges above the soil surface. At this heterotrophic stage, drastic changes may occur in the level of carbohydrates in the embryo axes of the germinating seeds. The application of this model system was aimed at comparing two situations during heterotrophic stages of seed germination. The first one is the stage when embryo axes are provided with an endogenous pool of soluble sugars due to the appropriately progressing mobilization of reserve nutrients in cotyledons, and the second stage is marked by a deficit of carbohydrates, so-called sugar starvation, resulting from disturbances in the mobilization of reserve substances. The experimental system, i.e., the embryo axes cultured *in vitro*, is a valuable model system, resembling natural growth conditions. The applied experimental system provides a unique possibility to study the direct effect of sugars on the pathogenicity of *F. oxysporum* f. sp. *lupini*. Samples were collected for analyses at 0 h and after 72 and 96 h of culture and frozen at −80 °C to determine the contents of ergosterol and moniliformin.

### 4.2. Preparation of Spore Suspension and Inoculation

*F*. *oxysporum* f. sp. *lupini* strain K-1018 was purchased from the Collection of Plant Pathogenic Fungi, Institute of Plant Protection-National Research Institute, Poznań. The fungus was maintained in the dark at 25 °C in Petri dishes (diameter 9 cm) on a potato dextrose agar (pH 5.5). After three weeks of its growth (*F*. *oxysporum*), a spore suspension was made [27,28,29,30,32,33,34,38]. The suspension containing spores was prepared by washing the *F*. *oxysporum* mycelium with sterile water and shook with glass pearls. Then the number of spores was counted using a hemocytometer chamber (Bürker, Labart, Gdańsk, Poland). The embryo axes were inoculated with the spore suspension at a concentration of 5 × 10^6^ spores per one mL. The inoculation was made by injecting 10 µL of spore suspension into the upper part of the embryo axes and additionally also by spraying the upper part of the embryo axes with the *F*. *oxysporum* inoculum.

### 4.3. Analysis of the Impact of Soluble Carbohydrates on Mycelium Growth and Sporulation

*F*. *oxysporum* f. sp. *lupini* strain K-1018 stock culture was grown on water agar (WA) in the dark for seven days at 25 °C. Thereafter, the following variants were applied: 1. WA as control, 2. WA with 60 mM sucrose, 3. WA with 120 mM glucose, 4. WA with 120 mM fructose. Next, the prepared media were melted and poured into 9 cm Petri dishes (10 mL/dish). After solidification, the growth media were inoculated at the center of each Petri dish with 5 mm plugs cuts with cork-borer from vigorously growing mycelial stock culture. The agar plates were then incubated at 25 °C in the dark. The growth of *F*. *oxysporum* mycelium was measured (mm) every two days. The test was completed when *F*. *oxysporum* completely filled the 9 cm diameter of the Petri dish. The sporulation was assessed after 21 days of incubation. The number of spores (macroconidia and microconidia) was determined with a Bürker hematological chamber (Labart). Preparation of spore suspension for counting was performed by flooding a single colony on a dish with 5 mL distilled water and scraping the surface of the fungal colony. Then the fungal spore suspension was harvested and transferred to 5 mL tubes. After agitation, a drop of the suspension was placed on a hematological chamber, and counting was performed by means of an optical microscope. The fungus’ morphological characteristics after treatment with soluble carbohydrates were determined after 21 days of incubation. Description consisted of: (i) form (the basic shape of the colony), (ii) elevation from both sides of dish, (iii) margin/border (edge of a colony), (iv) surface (colony appearance), (v) color (pigmentation). Each sugar was tested in ten replications.

### 4.4. Determination of Ergosterol (ERG)

The ergosterol (ERG) content, a chemical marker of the presence of fungus [67], was assayed, according to Waśkiewicz et al. [24]. Plant samples (100 mg) were suspended in 2 mL methanol in a culture tube, treated with 0.5 mL of 2 M aqueous sodium hydroxide, and sealed tightly. Samples were irradiated twice in a microwave oven (370 W) for 20 s. After 15 min, the contents of the culture tubes were neutralized with 1 M aqueous hydrochloric acid, then 2 mL methanol was added, and samples were extracted with *n*-pentane (3 × 4 mL). The combined pentane extracts were evaporated to dryness in a stream of nitrogen, and before analysis, dissolved in 1 mL of methanol, and 20 μL of the thus prepared mixture were analyzed by the high-performance liquid chromatography (HPLC). The ERG separation was performed on a 3.9 × 150 mm Nova Pak C-18, 4 µm column with methanol: acetonitrile (90:10, *v*/*v*) as the mobile phase at a flow rate of 1 mL min^−1^. EGR was quantified by high-performance liquid chromatography (HPLC) using a Waters 2695 apparatus (Waters Division of Millipore, Milford, MA, USA) with a Waters 2996 Array Detector set at 282 nm. The quantification of ERG was confirmed by a comparison of retention times with the external standard (ERG methanolic standard stock solution) and by co-injection of every tenth sample with an ERG standard. The correlation coefficient for ERG was 0.9997, with recovery 96%. The detection limit was 0.1 ng g^−1^, and the standard deviation was below 7%. All samples were injected in triplicate.

### 4.5. Determination of Moniliformin (MON)

The embryo axes (0.5 g) of each variant were homogenized with 75 mL of acetonitrile-methanol-water (16:3:1, *v*/*v*/*v*) and filtered (Whatman no. 4 filter paper). Moniliformin (MON) was extracted and purified according to the methods described by Waśkiewicz et al. [24]. MON was quantified by high-performance liquid chromatography (HPLC) using a Waters 2695 apparatus (Waters, Milford, MA, USA) with a C-18 Nova Pak column (3.9 × 300 mm, 4 µm) and a Waters 2996 Array Detector (λ_max_ = 229 nm). Acetonitrile: water (15:85, *v*/*v*) buffered with 10 mL of 0.1 M K_2_HPO_4_ in 40% *t*-butyl-ammonium hydroxide in 1 L of solvent used as the mobile phase (flow rate  =  0.6 mL min^−1^) [68]. Quantification of MON was performed by measuring the peak areas at the MON retention time according to the relevant calibration curve (the correlation coefficient for MON was 0.9990). The recovery for MON was 90%, the limit of detection was 1.0 ng g^−1,^ and the standard deviation was below 7%. All samples were injected in triplicate.

### 4.6. Analysis of the Impact of Soluble Carbohydrates and F. oxysporum f. sp. lupini Infection on the Growth of Yellow Lupine Embryo Axes

Biometric measurements of the length and fresh weight of embryo axes were performed for at least 10–20 axes and expressed in mm per organ and g per organ, respectively.

### 4.7. Statistics

Assays were conducted within three independent biological experiments. The normality of the distributions was tested using Shapiro-Wilk’s test [69]. The arithmetical means and standard deviations were calculated. The elementary comparisons between means were estimated with the two-sample *t*-test at the 0.05 level. To account for multiple testing, we used the Bonferroni correction. The relationships between observed traits were assessed on the basis of Pearson’s correlation and presented in the form of heatmaps. All calculations were conducted using the GenStat v. 18 statistical software package. A summary of the statistical significance of differences between the average values of each pair of parameters at *p* < 0.05 can be found in the Supplementary Files.

## Supplementary Materials

The following are available online at https://www.mdpi.com/1422-0067/21/19/7258/s1. Table S1: Statistical significance of differences between the average values of each pairs and Student’s *t*-test for the mycelium growth of *Fusarium oxysporum* f. sp. *lupini*. Table S2: Statistical significance of differences between the average values of each pairs and Student’s *t*-test for the sporulation of *Fusarium oxysporum* f. sp. *lupini*. Table S3: Statistical significance of differences between the average values of each pairs and Student’s *t*-test for the moniliformin and ergosterol contents in embryo axes of *Lupinus luteus* infected with *Fusarium oxysporum* f. sp. *lupini*. Table S4: Statistical significance of differences between the average values of each pairs and Student’s *t*-test for the fresh weight and length of *Lupinus luteus* embryo axes infected with *Fusarium oxysporum* f. sp. *lupini*.

## Abbreviations

+Sn	non-inoculated embryo axes and cultured in vitro on Heller’s medium with 60 mM sucrose
+Gn	non-inoculated embryo axes and cultured in vitro on medium with 120 mM glucose
+Fn	non-inoculated embryo axes and cultured in vitro on medium with 120 mM fructose
−Sn	non-inoculated cultured in vitro on medium without sucrose
+Si	inoculated and cultured with 60 mM sucrose
+Gi	inoculated and cultured with 120 mM glucose
+Fi	inoculated and cultured with 120 mM fructose
−Si	inoculated and cultured without sucrose
ERG	ergosterol
MON	moniliformin
WA	water agar
