# Supplementary material for: The Role of Saccharides in the Mechanisms of Pathogenicity of Fusarium oxysporum f. sp. lupini in Yellow Lupine (Lupinus luteus L.)
Source: Int J Mol Sci. 2020 Oct 1;21(19):7258. doi: 10.3390/ijms21197258 (PMC7582877; doi:10.3390/ijms21197258)
Supplement: Supplementary file 1 [file ijms-21-07258-s001.pdf]

Table S1. Statistical significance of differences between the average values of each pairs and Student's t-test for the mycelium growth of *Fusarium oxysporum* f. sp. *lupini*.

	Contrast	Mycelium growth
**Day 1**	Control: 60 mM sucrose	-0.245***
	Control: 120 mM glucose	-0.200***
	Control: 120 mM fructose	0.237***
	60 mM sucrose: 120 mM glucose	0.045
	60 mM sucrose: 120 mM fructose	0.482***
	120 mM glucose: 120 mM fructose	0.437***
**Day 2**	Control: 60 mM sucrose	-0.210***
	Control: 120 mM glucose	-0.110*
	Control: 120 mM fructose	0.459***
	60 mM sucrose: 120 mM glucose	0.100*
	60 mM sucrose: 120 mM fructose	0.669***
	120 mM glucose: 120 mM fructose	0.569***
**Day 3**	Control: 60 mM sucrose	-0.460***
	Control: 120 mM glucose	-0.290***
	Control: 120 mM fructose	0.672***
	60 mM sucrose: 120 mM glucose	0.170*
	60 mM sucrose: 120 mM fructose	1.132***
	120 mM glucose: 120 mM fructose	0.962***
**Day 4**	Control: 60 mM sucrose	-0.84***
	Control: 120 mM glucose	-0.50***
	Control: 120 mM fructose	0.92***
	60 mM sucrose: 120 mM glucose	0.34**
	60 mM sucrose: 120 mM fructose	1.75***
	120 mM glucose: 120 mM fructose	1.41***
**Day 5**	Control: 60 mM sucrose	-0.95***
	Control: 120 mM glucose	-0.66***
	Control: 120 mM fructose	1.09***
	60 mM sucrose: 120 mM glucose	0.28
	60 mM sucrose: 120 mM fructose	2.04***
	120 mM glucose: 120 mM fructose	1.75***
**Day 6**	Control: 60 mM sucrose	-0.83***
	Control: 120 mM glucose	-0.77***
	Control: 120 mM fructose	1.25***
	60 mM sucrose: 120 mM glucose	0.06
	60 mM sucrose: 120 mM fructose	2.07***
	120 mM glucose: 120 mM fructose	2.01***
**Day 7**	Control: 60 mM sucrose	-0.3
	Control: 120 mM glucose	-0.26
	Control: 120 mM fructose	1.36***
	60 mM sucrose: 120 mM glucose	0.04
	60 mM sucrose: 120 mM fructose	1.66***
	120 mM glucose: 120 mM fructose	1.62***
*** P<0.05; ** P<0.01; *** P<0.001**		

Table S2. Statistical significance of differences between the average values of each pairs and Student's t-test for the sporulation of *Fusarium oxysporum* f. sp. *lupini*.

	Contrast	Sporulation
**Day 21**	Control: 60 mM sucrose	0.355***
	Control: 120 mM glucose	0.920***
	Control: 120 mM fructose	0.585***
	60 mM sucrose: 120 mM glucose	0.565***
	60 mM sucrose: 120 mM fructose	0.230**
	120 mM glucose: 120 mM fructose	-0.335***
**** P<0.01; *** P<0.001**		

Table S3. Statistical significance of differences between the average values of each pairs and Student's t-test for the moniliformin and ergosterol contents in embryo axes of *Lupinus luteus* infected with *Fusarium oxysporum* f. sp. *lupini*.

Time (h)	Contrast	Moniliformin content	Ergosterol content
**0**	0n: 0i	-1	-3.05**
**24**	+Sn: +Si	-3.1	-8.25***
	+Gn: +Gi	-9.2	-6.38***
	+Fn: +Fi	-7.8	-1.68
	−Sn: −Si	-35.4***	-14.92***
	+Sn: +Gn	0	0
	+Sn: +Fn	0	0
	+Gn: +Fn	0	0
	+Sn: −Sn	0	0
	+Si: −Si	-32.3**	-6.67***
	+Gi: −Si	-26.2*	-8.54***
	+Fi: −Si	-27.6**	-13.24***
	+Si: +Gi	-6.1	1.87*
	+Si: +Fi	-4.6	6.58***
	+Gi: +Fi	1.5	4.71***
**48**	+Sn: +Si	-3.7	-2.54**
	+Gn: +Gi	-4.3	-0.78
	+Fn: +Fi	-12.6	-0.4
	−Sn: −Si	-228.1***	-8.83***
	+Sn: +Gn	0	0
	+Sn: +Fn	0	0.03
	+Gn: +Fn	0	0.03
	+Sn: −Sn	0	0.24
	+Si: −Si	-224.4***	-6.05***
	+Gi: −Si	-223.8***	-7.81***
	+Fi: −Si	-215.5***	-8.23***
	+Si: +Gi	-0.6	1.76
	+Si: +Fi	-8.8	2.18*
	+Gi: +Fi	-8.3	0.42
**72**	+Sn: +Si	-1	-3.05**
	+Gn: +Gi	-3.1	-8.25***

	+Fn: +Fi	-9.2	-6.38***
	-Sn: -Si	-7.8	-1.68
	+Sn: +Gn	-35.4***	-14.92***
	+Sn: +Fn	0	0
	+Gn: +Fn	0	0
	+Sn: −Sn	0	0
	+Si: −Si	0	0
	+Gi: −Si	-32.3**	-6.67***
	+Fi: −Si	-26.2*	-8.54***
	+Si: +Gi	-27.6**	-13.24***
	+Si: +Fi	-6.1	1.87*
	+Gi: +Fi	-4.6	6.58***
**96**	+Sn: +Si	1.5	4.71***
	+Gn: +Gi	-3.7	-2.54**
	+Fn: +Fi	-4.3	-0.78
	−Sn: −Si	-12.6	-0.4
	+Sn: +Gn	-228.1***	-8.83***
	+Sn: +Fn	0	0
	+Gn: +Fn	0	0.03
	+Sn: −Sn	0	0.03
	+Si: −Si	0	0.24
	+Gi: −Si	-224.4***	-6.05***
	+Fi: −Si	-223.8***	-7.81***
	+Si: +Gi	-215.5***	-8.23***
	+Si: +Fi	-0.6	1.76
	+Gi: +Fi	-8.8	2.18*
*** P<0.05; ** P<0.01; *** P<0.001**			

Table S4. Statistical significance of differences between the average values of each pairs and Student's t-test for the fresh weight and length of *Lupinus luteus* embryo axes infected with *Fusarium oxysporum* f. sp. *lupini*.

Time (h)	Contrast	Embryo axis fresh weight	Embryo axis lenght
**0**	0n: 0i	0.0002	0.36
**24**	+Sn: +Si	0.0085***	3.63***
	+Gn: +Gi	0.0065***	3.42***
	+Fn: +Fi	0.004**	2.5***
	−Sn: −Si	0.0087***	2.37***
	+Sn: +Gn	0.0015	-0.74**
	+Sn: +Fn	0.0045**	0.66*
	+Gn: +Fn	0.003*	1.4***
	+Sn: −Sn	0.002	2.53***
	+Si: −Si	0.0022	1.26***
	+Gi: −Si	0.0028	2.21***
	+Fi: −Si	0.0023	1.73***
	+Si: +Gi	-0.0005	-0.95***
	+Si: +Fi	0	-0.47
	+Gi: +Fi	0.0005	0.48
**48**	+Sn: +Si	0.0177***	11.11***
	+Gn: +Gi	0.02***	7.67***

	+Fn: +Fi	0.0223***	11.5***
	−Sn: −Si	0.0197***	5.79***
	+Sn: +Gn	0.0043**	0.79**
	+Sn: +Fn	0.0051***	-0.23
	+Gn: +Fn	0.0008	-1.02***
	+Sn: −Sn	0.0092***	7.32***
	+Si: −Si	0.0112***	2***
	+Gi: −Si	0.0048**	4.65***
	+Fi: −Si	0.0016	1.84***
	+Si: +Gi	0.0065***	-2.65***
	+Si: +Fi	0.0097***	0.16
	+Gi: +Fi	0.0032*	2.82***
**72**	+Sn: +Si	0.031***	14.26***
	+Gn: +Gi	0.05***	21.67***
	+Fn: +Fi	0.0273***	11.8***
	-Sn: -Si	0.0417***	10.84***
	+Sn: +Gn	-0.0132***	-7.04***
	+Sn: +Fn	0.0076***	2.89***
	+Gn: +Fn	0.0208***	9.93***
	+Sn: −Sn	0.007***	9.74***
	+Si: −Si	0.0177***	6.32***
	+Gi: −Si	0.012***	5.96***
	+Fi: −Si	0.0138***	5.89***
	+Si: +Gi	0.0057***	0.36
	+Si: +Fi	0.0039*	0.43
	+Gi: +Fi	-0.0018	0.07
**96**	+Sn: +Si	0.0627***	18.37***
	+Gn: +Gi	0.0904***	34.75***
	+Fn: +Fi	0.0709***	31.7***
	−Sn: −Si	0.0517***	15.53***
	+Sn: +Gn	-0.0242***	-15.5***
	+Sn: +Fn	-0.0114***	-13.92***
	+Gn: +Fn	0.0128***	1.59***
	+Sn: −Sn	0.0305***	10.84***
	+Si: −Si	0.0195***	8***
	+Gi: −Si	0.016***	7.12***
	+Fi: −Si	0.0227***	8.59***
	+Si: +Gi	0.0035*	0.88**
	+Si: +Fi	-0.0032*	-0.59*
	+Gi: +Fi	-0.0067***	-1.47***
*** P<0.05; ** P<0.01; *** P<0.001**			
